# Efficacy of Curcumin Gel on Zinc, Magnesium, Copper, IL-1*β*, and TNF-*α* in Chronic Periodontitis Patients

**DOI:** 10.1155/2020/8850926

**Published:** 2020-10-05

**Authors:** Chenar Anwar Mohammad

**Affiliations:** Department of Periodontology, College of Dentistry, Hawler Medical University, Erbil, Kurdistan Region, Iraq

## Abstract

Curcumin exhibits antibacterial, antioxidant, and anti-inflammatory effects and has been suggested as a treatment for inflammatory diseases. The study is aimed at evaluating the effect of curcumin gel on serum levels of micronutrients (zinc, copper, and magnesium) and proinflammatory cytokines (IL-1*β* and TNF-*α*) in chronic periodontitis patients. Ninety subjects with an age of 25-54 were included in this study. From the total number, 30 subjects with healthy periodontium (control group) (mean age = 37.30 ± 7.08) were employed for the sole purpose of obtaining the normal mean values of clinical, chemical, and immunological parameters, and 60 with chronic periodontitis (mean age = 36.73 ± 6.22) were divided randomly into 2 groups, of which each group included 30 subjects. Group A received scaling and root planing SRP and curcumin gel injection covered by Coe pack for 7 days, and group B received SRP alone covered by Coe pack. Clinical parameters (plaque index, gingival index, bleeding on probing, pocket depth, and clinical attachment loss measurements) and blood samples were collected before and after 1 month of treatment to measure serum levels of zinc, copper, magnesium, IL-1*β*, and TNF-*α*. The results showed significant micronutrient alteration and increase of proinflammatory cytokines in the chronic periodontitis group as compared to healthy control (*P* ≤ 0.05), and curcumin gel had a significant effect on the reduction of IL-1*β*, TNF-*α*, copper, and clinical parameters (*P* ≤ 0.05) and increase of zinc and magnesium levels after 1 month as compared to baseline (*P* ≤ 0.05), nearly the same pattern for group B but with nonsignificant differences for Zn (*P* > 0.05). In conclusion, curcumin gel resulted in a more significant reduction in clinical parameters, inflammatory mediators, and copper and increase of zinc and magnesium levels as compared to SRP alone.

## 1. Introduction

Periodontitis (CP) is an inflammatory disease of the supporting tissues of the teeth, caused by pathogenic microorganisms, which may result in attachment loss and alveolar bone resorption through a specific elicited host inflammatory response [1].

During periodontitis, several inflammatory cytokines have been demonstrated to be linked to mechanisms that promote the progression of periodontal tissue destruction [2]. Of these, IL-1, IL-6, and IL-17 alone or in synergy with IL-1*β*, TNF-*α*, toll-like receptors, and prostaglandin E_2_ (PGE_2_) have been shown to stimulate gingival fibroblasts, epithelial cells, and macrophages to control homeostasis of periodontal tissues and to release proinflammatory mediators for tissue homeostasis [3, 4]. These inflammatory cytokines promote the expression of receptor activator of nuclear factor-kappa B (NF-*κ*B) ligand (RANKL) in the stromal cells and osteoblasts [5] that, together with osteoprotegerin, represents the key pathways that control alveolar bone resorption [6, 7].

Since periodontal tissue vitality in health and disease depends strongly on an adequate source of essential nutrients being available to the host, certain micronutrients such as zinc (Zn), magnesium (Mg), and copper (Cu) are essential for the normal metabolism of proteins, carbohydrates, and lipids. Zn is an integral component of antioxidant enzymes and participates in the synthesis and actions of the hormones, which are intimately linked to bone metabolism [8], and its altered levels can cause oxidative stress. It plays an important role in immune function, wound healing, protein synthesis, DNA synthesis, and cell division. Therefore, its deficiency can affect the functioning of immune cells such as monocytes, natural killer cells (reduced cytotoxicity), neutrophils (reduced phagocytosis), T cells, and lymphocytes (decreased apoptosis). Furthermore, it increases the secretion of proinflammatory cytokines and then bone resorption [9]. Mg is another micronutrient and acts as a critical cofactor involved in the carbohydrate, lipid, and protein metabolism; it is also a fundamental element of the structure of cellular and subcellular membranes with the major function of offering membrane stability [10, 11]. Both Zn and Mg are considered to be protective with their antioxidant properties [12]. Imbalance of Zn and copper (Cu) in the serum can predispose an individual to the risk of developing chronic periodontitis [13].

Cu serves as a cofactor for metalloenzymes like superoxide dismutase, an essential antioxidant for chronic periodontitis, and it is essential for immunity and combating the oxidative stress induced by reactive oxygen and nitrogen species. The optimum levels of Cu are essential for preventing exacerbation of inflammatory pathways [13] and for proper connective tissue development, while elevated levels of Cu in the serum of periodontitis patients can cause certain alterations in collagen metabolism and then connective tissue destruction [14]. Therefore, metabolic alteration of micronutrient levels of Zn, Mg, and Cu in serum can contribute to the progression and increased risk of developing chronic periodontitis due to decreased regenerative capacity and impaired immune function, along with the development of excessive oxidative stress.

The long-term success in the therapy of periodontitis changes mostly on the efficient removal of bacterial biofilms and its related endotoxins that are deposited at supra- and subgingival levels, eliciting a specific inflammatory response that determines periodontal tissue destruction [15, 16]. Nonsurgical periodontal treatment by mechanical instrumentation, which is aimed at reducing pathogenic periodontal pathogens in biofilms, has been demonstrated to have limited long-term effects [3]. Although meta-analysis has highlighted an improvement in periodontal parameters [17], some reports conversely demonstrated that SRP alone does not allow complete removal of periodontal pathogens, particularly in deep periodontal pockets, because it does not eradicate all bacteria and their inflammatory mediators' well [18]. In fact, it was reported that the persistence of periodontal inflammation could lead to residual pocketing and the constant presence of periodontal bacteria which, in turn, rebound tissue destruction [19].

Therefore, deep periodontal pockets require an adjunctive antimicrobial regimen to aid in the mechanical debridement of tooth surfaces by local and systemic delivery of chemical agents. Local drug delivery of agents in periodontal pockets has the benefit of maintaining an adequate concentration of the drugs at the target site while minimizing the exposure of the total body to the drug. Herbal plants are used as an alternative to systemic agents. Because of the relatively safe nature of herbal extracts, many herbal products and its components are used in periodontics as local drug delivery. Considering the undesirable side effects and limitations of various synthetic agents, a wide range of herbal extracts such as *Acacia catechu*, *Aloe vera*, *Azadirachta indica*, *Glycyrrhiza glabra*, *Ocimum sanctum L.*, *Curcuma longa*, and *Matricaria chamomilla* containing active component has been shown potent anti-inflammatory, antibacterial, antioxidant, and astringent properties with significant improvement in gingival health when used subgingival irrigation. [20]. Turmeric (also known as Curcuma longa) is a member of the ginger family, Zingiberaceae [21], and curcumin (diferuloylmethane), the main yellow bioactive component of turmeric, has been shown to have a wide spectrum of biological actions. These include its anti-inflammatory, antibacterial, antivenom, antiulcer, enhancer of healing, and myogenesis properties [20].

Since SRP does not allow complete removal of periodontal pathogens, there is an increasing interest from several studies in therapies that could improve the effects of SRP as adjuvant therapies [20, 22, 23]. Therefore, the present study was aimed at investigating the hypothesis that curcumin gel could be effective in reducing periodontal parameters, controlling the levels of the inflammatory mediators, and balancing micronutrient serum levels when administrated locally.

## 2. Materials and Methods

### 2.1. Setting and Time of Study

The study was carried out in the periodontic and biochemical laboratories of the Basic Science Departments/College of Dentistry/Hawler Medical University/Erbil city, Iraq. The study period was from February 2019 to February 2020.

### 2.2. Study Group

The clinical comparative study was performed on 90 subjects who visited the Department of Periodontics, College of Dentistry, Hawler Medical University, Erbil, Iraq. Before starting the study, verbal and written informed consent was obtained from those who agreed to participate voluntarily in this study, and the study protocol was reviewed and approved by the Institutional Ethical Committee of the College of Dentistry, Hawler Medical University, Erbil, Iraq.

For all patients, the inclusion criteria were age ≥ 25 to 45 years, individuals with ≥20 teeth, systemically healthy controls, cooperative patients who could be motivated to comply with further oral hygiene instructions, and patients who consented to participate in the study while patients with a history of periodontal treatment or antibiotic drug intake during the last 6 months, pregnant, lactating or menopausal women, alcoholics, tobacco smokers, drug abusers, malnourished and on vitamin supplementations, and individuals who regularly used mouthwashes were excluded from the study. For chronic periodontitis, the inclusion criteria were as follows: probing pocket depth (PPD) ≥ 4 mm and clinical attachment level (CAL) ≥ 2 mm in at least 40% of the analyzed sites [16, 24], and no furcation involvement, while for healthy control patients, the inclusion criteria were as follows: PPD ≤ 3 mm, and no sites present with clinical attachment loss (CAL = 0) or bleeding on probing (BOP = 0).

From the total number, there are 30 subjects with an average age of 37.30 ± 7.08 and normal healthy periodontium (mean PI = 0.100 ± 0.079, GI = 0.070 ± 0.092, PPD = 1.583 ± 0.708, CAL = 0.00, and BOP = 0.00). They were employed as a control group to estimate the normal mean value of clinical, chemical, and immunological parameters only. The remaining 60 patients, with an average age of 36.73 ± 6.22 and chronic periodontitis, were divided randomly into 2 groups:

1. Group A, test group, included 30 subjects that received scaling and root planing and curcumin gel application (*C. longa* extract, 10 mg) (Curenext Oral Gel, Abbott Healthcare Limited, Mumbai, India) into the periodontal pocket for 5 min, and then, the area was covered by periodontal dressing (Coe pack)
2. Group B included 30 subjects that received SRP alone covered by Coe pack

Scaling and root planing were performed in one session, and the patients received single intrasulcular applications of curcumin gel into the periodontal pockets. The subgingival delivery of curcumin gel was performed with a 2 ml disposable syringe equipped with a blunted 25-gauge needle, which was bent along its shank by 130°, in a continuous procedure until the pocket was completely filled. Care was taken to apply the gel without causing trauma or damaging the periodontal tissues. After insertion of the local drug delivery system, the area was covered with a periodontal pack (Coe pack) for 7 days. Coe pack was used to cover the pocket, to prevent the ingress of oral fluids, and to ensure retention of the gel within the pocket.

Oral hygiene instructions were given to patients, including not to eat for at least one hour, not wash their mouth, not brush the treated areas for 12 hours, not use dental floss or interproximal cleaning devices for 10 days, and not use any mouthwash during the study. Subjects were recalled after 7 days for the removal of the periodontal dressing.

### 2.3. Periodontal Assessment

The clinical periodontal parameter examination was carried out for all participants by the same examiner to avoid subjective bias, at the baseline and after 1 month of periodontal therapy. The clinical periodontal parameters included are as follows: PPD was assessed by gently inserting a periodontal probe from the gingival margin to the base of the sulcus or pocket at four surfaces of each tooth [25], CAL was assessed by measuring the distance from the cementoenamel junction (CEJ) to the base of pocket by periodontal probe [25], PI was assessed by measuring the thickness of plaque for four surfaces of the examined tooth and given a score from 0 to 3 [26], gingival index (GI) was identified by measuring the extent and severity of gingival inflammation throughout an inspection by naked eyes and by gentle probing for four gingival surfaces of the examined tooth [27], BOP was performed by running a periodontal probe gently along the inner surface wall of the gingival sulcus, and bleeding was noted after 30 seconds [28]. The sites of the measurements were mesiobuccal/labial, distobuccal/labial, midbuccal/labial, and midpalatal/lingual for all teeth with the exception of all 3^rd^ molars, using a manual periodontal probe (PC-PUNC 15 Hu-Friday, Chicago, USA) for GI, BOP, PPD, and CAL assessments and straight explorer probe (DenMat, USA) for PI measurements. Patients with periodontal disease were under conventional periodontal treatment consisting of motivation, oral hygiene instructions, scaling (supra- and subgingival), and root planing.

### 2.4. Biochemical and Immunological Parameter Measurements

Zn, Mg, and Cu levels were measured using atomic absorption spectrophotometry. IL-1*β* and TNF-*α* levels were measured using enzyme-linked immunosorbent assay (indirect Elisa technique) supplied by a specific Elisa kit (KOMA BIOTECH INC., USA), and the procedure was performed according to the manufacturer's instructions.

### 2.5. Blood Sample

A 5 ml sample of venous blood was drawn from the subject through a disposable syringe and transferred to a centrifuge tube. The sample was centrifuged at 3000 rpm for 15 min to obtain a supernatant. Then, the supernatant was aspirated and collected in separate test tubes to measure the serum levels of Zn, Mg, Cu, IL-1*β*, and TNF-*α* in healthy control and in chronic periodontitis patients at baseline before therapy and after 1 month of therapy.

### 2.6. Statistical Analysis

Data were presented as number, mean, degree of freedom, and standard deviation. The data were statistically analyzed using the Statistical Package for Science Services (SPSS, version 22). Student's *t*-test (paired and unpaired *t*-test) was used for the comparison between the studied groups. *P* ≤ 0.05 was accepted as statistically significant.

## 3. Results

Ninety subjects comprising 55 females (61.1%) and 35 males (39.9%) with the mean age of 37.06 ± 6.50 years participated in this study. The largest proportion of the subjects (67.8%) ranged in age from 35 to 44 years, 21.1% ranged from 25 to 34 years, and the lowest proportion (11.1%) ranged from 45 to 54 years, as shown in Table 1. In the healthy control group, 17 subjects (56.7%) were female and 13 (43.3%) were male, with the mean age of 37.30 ± 7.08 years, and in the chronic periodontitis group, 38 subjects (63.3%) were female and 22 (36.7%) were male, with the mean age of 36.73 ± 6.22 years.

Regarding clinical results, Table 2 shows that in group A and after curcumin gel application, the mean value of the PI score reduced significantly from 2.127 ± 0.177 at baseline before therapy to 0.533 ± 0.184 after 1 month of treatment. There were also significant reductions in the mean values of GI and BOP after 1 month of treatment (0.618 ± 0.081 and 20.847 ± 3.038%, respectively) as compared to their mean values at baseline before treatment (2.103 ± 0.133 and 70.869 ± 4.950%, respectively, *P* ≤ 0.05). Finally, the results also showed a significant reduction in the mean values of PPD and CAL from 4.650 ± 0.544 mm and 5.250 ± 0.469 mm, respectively, at baseline to 3.067 ± 0.173 mm and 3.583 ± 0.265, respectively, after therapy (*P* ≤ 0.05). Nearly the same pattern emerged for group B, the results showing significant reductions in the mean values of PI, GI, BOP, PPD, and CAL from 2.017 ± 0.102, 2.047 ± 0.183, 68.161 ± 4.075%, 3.233 ± 0.254 mm, and 5.100 ± 0.254 mm, respectively, at baseline before SRP to 0.693 ± 0.141, 0.925 ± 0.104, 31.547 ± 4.075%, 4.617 ± 0.254 mm, and 4.067 ± 0.254 mm, respectively, after 1 month of SRP (*P* ≤ 0.05).

Regarding chemical parameters, Table 3 shows significant increases in the mean serum levels of Zn and Mg after 1 month of curcumin gel application in group A (171.273 ± 61.448 *μ*g/dl and 1.8277 ± 0.087 mg/dl, respectively) as compared to baseline (130.532 ± 33.892*μ*g/dl and 1.540 ± 0.081 mg/dl, respectively) before therapy (*P* ≤ 0.05) and significant reduction of the mean serum level of Cu from 199.364 ± 32.004 *μ*g/dl at baseline to 178.747 ± 31.504 *μ*g/dl after curcumin gel treatment (*P* < 0.05). For group B, the results show a significant increase in the mean serum level of Mg after 1 month of SRP treatment (1.607 ± 0.058 mg/dl) as compared to baseline (1.540 ± 0.058 *μ*g/dl) (*P* ≤ 0.05), nonsignificant increase in the mean serum level of Zn after 1 month of treatment (97.897 ± 14.952 *μ*g/dl) as compared to baseline (97.667 ± 6.031 *μ*g/dl) (*P* > 0.05), and significant reduction of the mean serum level of Cu from 186.577 ± 34.583 *μ*g/dl to 159.444 ± 12.792 *μ*g/dl after treatment (*P* ≤ 0.05). Additionally, Table 4 shows a significant reduction in the mean serum values of IL-1*β* and TNF-*α* in group A (3.450 ± 0.486 pg/ml and 7.374 ± 0.652 pg/ml, respectively) and group B (4.534 ± 0.529 pg/ml and 8.483 ± 0.453 pg/ml, respectively) after 1 month of treatment as compared to baseline before treatment in group A (5.036 ± 0.609 pg/ml and 10.561 ± 0.713 pg/ml, respectively) and group B (10.561 ± 0.606 pg/ml and 10.519 ± 0.717 pg/ml, respectively, *P* ≤ 0.05).

Based on a comparison between the curcumin group (group A) and the SRP group (group B) after 1 month of therapy, Table 5 shows significant differences between the two groups in regard to clinical periodontal, biochemical, and immunological parameters (*P* ≤ 0.05). Slightly more reductions were seen in the mean values of PI, GI, POP, PPD, CAL, IL-1*β*, and TNF-*α* in group A as compared to group B and more significant increases in the mean serum levels of Zn and Mg in group A as compared to group B.

Based on a comparison between healthy control and chronic periodontitis patients (A and B groups) at baseline before therapy, Table 6 shows significant increase in the mean values of clinical periodontal parameters PI, GI, BOP, PPD, and CAL in chronic periodontitis patients in both A and B test groups as compared to the control group. The results for biochemical parameters also show a significant increase in the mean serum levels of Zn and Cu in chronic periodontitis patients in both A and B groups as compared to control, while the mean level of serum Mg was significantly lower in chronic periodontitis patients in both A and B groups as compared to the control group (group C) (*P* ≤ 0.05). Finally, Table 6 also shows that the mean IL-1 beta and TNF-alpha level values were significantly increased in chronic periodontitis patients as compared to control (*P* ≤ 0.05). Based on a comparison between healthy control (group C) and chronic periodontitis patients of both groups after 1 month of periodontal therapy, Table 7 shows significant differences between control and group A in regard to PI, GI, PPD, BOP, CAL, Zn, Mg, Cu, and IL-1 beta with the exception of TNF-alpha (*P* = 0.327), with the same applying for group B with the exception of Zn (*P* = 0.131).

## 4. Discussion

The results showed a significant increase in clinical periodontal parameters, inflammatory mediators, and micronutrient serum level alteration in chronic periodontitis. Moreover, curcumin gel application resulted in a significant reduction of clinical periodontal parameters, and inflammatory mediators with significant improvement of micronutrient serum level, nearly the same pattern for SRP alone. Also, curcumin gel resulted in a more significant reduction of clinical, inflammatory mediators, and improvement of micronutrient level as compared to SRP alone.

Micronutrients such as Zn, Cu, and Mg play an important role to maintain adequate immune response as well as to combat oxidative stress. Inflammatory stimuli from dental plaque promote the release of reactive free radicals and also exhibit metabolic changes that are modulated by potent soluble proinflammatory mediators known as cytokines. Many studies have assessed the levels of micronutrients and proinflammatory cytokines in chronic periodontitis patients. However, this is the first study to explore the effect of local delivery of curcumin gel after SRP on the serum levels of micronutrients and proinflammatory cytokines in chronic periodontitis patients, in addition to evaluating curcumin's effect clinically.

Clinically, the present study revealed significant improvements in clinical periodontal parameters and healing of periodontal tissue after curcumin gel application in group A and after SRP in group B, through their significant effect on reduction of the mean values of PI, GI, BOP, PPD, and gain in clinical attachment level after 1 month of treatment as compared to baseline. Although there were highly significant differences between A and B groups after 1 month of treatment, slightly more reductions of PI, GI, BOP, and gain of CAL were seen after curcumin gel application. The reduction of gingival inflammation and bleeding on probing may be attributed to the anti-inflammatory and antioxidant properties of curcumin through inhibition of NF-*κ*B activation [29–31] and downregulation of proinflammatory enzyme cyclooxygenase-2 [30, 31] by reducing the inflammatory mediators generated via the arachidonic acid pathway [32], which causes shrinkage by reducing inflammatory edema and vascular engorgement of connective tissue [33, 34], which promote migration of various cells including fibroblasts into the wound bed, thus resulting in a reduction in vascularization by fibrosis of the connective tissue [32] which enhances wound healing by causing an increase in the number of fibroblasts [35]. Also, the slightly more reduction of PI and PPD after curcumin gel application may be due to curcumin's antiplaque and antimicrobial properties [36, 37]. Gopinath et al. [38] showed that when curcumin is incorporated into collagen, it acts as a supportive matrix for slow release, increases wound reduction, and enhances cellular proliferation. Curcumin enhances wound healing by causing an increase in fibronectin and promotes migration of epithelial cells to wounded sites by promoting localization of TGF-*β*1, thus helping reepithelization [36].

The results are in the same line with Bhatia et al. who treated 25 patients with chronic periodontitis with PPD of at least 5 mm in depth. The test group received SRP along with intrapocket application of a gel containing 1% curcumin at baseline and at 1-, 3-, and 6-month intervals [20]. The control group received SRP alone. At the ends of the observation period, there were significant improvements in the clinical parameters, including reduction in PPD and bleeding, and gain in CAL in both groups but with more pronounced improvement in the test group. In regard to the microbiological parameters, curcumin significantly reduced the levels of *P. gingivalis*, *P. intermedia*, *F. nucleatum*, and *Capnocytophaga* sp. at the end of the six-month observation period [20]. Additionally, in a clinical trial of 30 patients comparing 1% curcumin gel and 0.1% chlorhexidine gel application, following SRP, significant improvement in the clinical parameters as well as reduction of the colony-forming units was reported in both groups. However, more significant reduction was noted in the curcumin group and the authors recommended its use over chlorhexidine, especially because of its minimal side effects [39].

In the present study, we chose 2 important proinflammatory mediators (TNF-*α* and IL-1*β*) to examine the effect of curcumin gel on the production of these cytokines, since these cytokines participate to various extents in the production and the development of inflammation through recruitment and activation of inflammatory cells. They are actively involved in jeopardizing periodontal tissues by affecting the activities of leukocytes, osteoclasts, and MMPs to mediate alveolar bone resorption and collagen destruction [40].

For immunological parameters, the result showed a significant increase in serum mean levels of Il-1*β* and TNF-*α* in chronic periodontitis patients (A and B groups) as compared to healthy control subjects. After 1 month of treatment, a significant reduction of IL-1 beta and TNF-alpha was seen in both A and B groups, but with highly significant differences between the two groups, since slightly more reductions of IL-1 beta and TNF-alpha were seen after curcumin gel application. These reductions may be due to curcumin's effective control of IL-1 beta, TNF-alpha expression, and PGE2 synthesis by modulating NF-*κ*B activation. The anti-inflammatory actions of curcumin may be attributed to several mechanisms. Firstly, it suppresses the activation of the transcription factor NF-*κ*B [41], since stimulation of cells with various inflammatory agents such as TNF-*α*, IL1*β*, and LPS from gram-negative periodontal pathogens leads to activation and transcription of NF-*κ*B [42] and curcumin has been shown to suppress this activation process [43]. Secondly, curcumin downregulates the expression of cyclooxygenase-2, an enzyme that catalyzes the synthesis of prostaglandins (PGs) which is linked to most forms of inflammation, including periodontitis, and curcumin significantly inhibits LPS-induced COX-2 expression, contributing to a decreased synthesis of PGE2, which is a potent stimulator of bone resorption [44]. Another molecular target of curcumin is inducible nitric oxide synthase (iNOS), which is a strong proinflammatory molecule that is regulated by NF-*κ*B. IL-1*β*, TNF-*α*, and bacterial LPS increase iNOS expression, indicating that iNOS may also play a role in bone inflammation. Curcumin inhibits the synthesis of iNOS protein [45]. Also, curcumin downregulates the expression of various vascular endothelial cell surface adhesion molecules that are important in inflammation because of their ability to facilitate leukocyte extravasation from the vasculature into the tissues. Cell adhesion proteins are not normally present on the endothelial cell surface but are induced by various proinflammatory cytokines such as IL-1*β* and TNF-*α* [46], and curcumin downregulates the levels of cell adhesion molecules through its inhibition of NF-*κ*B and subsequent inhibition of proinflammatory cytokines IL-1*β* and TNF-*α* that stimulate their expression. Additionally, curcumin inhibits the expression of the transcription factor AP-1, and by inhibiting the AP-1 pathway, curcumin inhibits the expression of inflammatory mediators/cytokines, iNOS, and several MMP 1-3, -9, 14, and 13 secretions [47, 48].

Chemically, the present study showed that the serum mean levels of Mg, Cu, and Zn were significantly altered in chronic periodontal patients (increased mean levels of Cu and decreased mean levels of Zn and Mg) in both A and B groups as compared to healthy control, and after 1 month of curcumin gel application, significant increases in the mean levels of Zn and Mg and decrease of Cu level were observed. Nearly the same pattern was found in group B, but with a nonsignificant difference for Zn after 1 month of therapy. Furthermore, in a comparison between A and B groups, highly significant differences were found. The imbalance in the micronutrient levels of Zn, Mg, and Cu in serum can predispose an individual to the risk of developing periodontitis, and this may be due to decreased regenerative capacity and impaired immune function along with the development of excessive oxidative stress.

The significant alteration of serum Zn level in chronic periodontitis patients for both A and B groups is in the same line with studies performed by Pushparani et al. [49], Pushparani [50], and Pushparani [51] which showed increased serum zinc levels in CP cases as compared to the healthy control group. To the contrary, Thomas et al. [13, 52] carried out studies to evaluate the serum levels of zinc, copper, and iron in periodontitis cases with and without type 2 diabetes mellitus, and the results showed that the serum zinc levels were lower in CP cases as compared to healthy control. They associated zinc deficiencies and increased levels of copper and iron with increased oxidative stress along with an altered immune response which could lead to various diabetic complications including periodontitis [13]. Regarding Mg, the present study found decreased Mg serum levels in chronic periodontitis cases as compared to healthy control, and this may be due to the elevation of oxidative stress generation in CP, so when the oxidative stress is increased, the Mg levels in the body may be depleted [53]. The results of the present study for serum Mg levels were in the same line with the studies carried out by Pushparani et al. [49] and Agrawal et al. [53] considering CP and healthy control groups only. In their study, they observed that Mg concentration decreased slightly but this was nonsignificant in CP cases. They found that as the disease progressed, Mg levels were decreased. Pushparani et al. [49] evaluated serum concentrations of zinc and magnesium in type 2 diabetes patients with periodontitis. They observed that as the disease progressed with CP, the Mg level decreased more significantly, indicating that diabetic individuals who develop periodontitis are more prone to diabetic complications. The present study observed a significant increase in serum mean levels of Mg and Zn after curcumin gel application, with nearly the same elevation in group B after SRP but with nonsignificant differences compared to baseline for Mg. This may be due to that curcumin gel can improve the serum levels of Mg and Zn as well as improving periodontal health

For Cu, the present study revealed a significant increase in the mean serum level of Cu in chronic periodontitis patients of both A and B groups and significant improvement through its reduction after treatment in both A and B groups. The elevation in serum Cu level can cause certain alterations in periodontal collagen metabolism and hence can promote periodontitis. The results of the present study are in the same line with Thomas et al. [13] who reported elevation of serum Cu level in periodontitis patients that caused certain alterations in collagen metabolism.

Sundaram et al. [54] also demonstrated the levels of Cu in diabetes and nondiabetes patients with chronic periodontitis; they reported that Cu levels were elevated at baseline and were improved significantly 3 months after nonsurgical periodontal therapy, even in those participants with uncontrolled type 2 diabetes mellitus. Additionally, Turnlund [55] reported that the increase in serum Cu can modulate immune function and antioxidant status. The study found that the increase in Cu levels can have an impact on the immune system, including neutrophil numbers, lymphocyte proliferation, and antigen-specific antibody production [55]. In the present study, the reduction of serum Cu level, an increase of Zn and Mg, and a decrease of Il-1*β* and TNF-*α* were observed after curcumin gel application, indicating an improvement in the periodontal status after curcumin gel application. Nearly the same pattern was observed after treatment with SRP alone.

## 5. Conclusion

Metabolic alteration of micronutrient levels of Zn, Mg, and Cu in serum can contribute to the progression and increased risk of developing chronic periodontitis due to decreased regenerative capacity and impaired immune function (elevation of proinflammatory cytokine levels), along with the development of excessive oxidative stress. The local delivery of curcumin gel can improve the variation and serum level of micronutrients (through significant reduction of Cu level and increase of Zn and Mg levels) and proinflammatory cytokines (through significant reduction of IL-1*β* and TNF-*α*) and therefore improve periodontal health. Nearly the same pattern can be obtained after SRP treatment alone but with a high significant difference with curcumin gel application.

## Figures and Tables

**Table 1:** Age and gender distribution of study groups.

Variables	Levels	Healthy control		Periodontitis groups		All groups	
		No	%	No	%	No	%
Age	25-34	8	26.7%	11	18.3%	19	21.1%
	35-44	19	63.3%	42	70.0%	61	67.8%
	45-54	3	10.0%	7	11.7%	10	11.1%
	Total	30	100%	60	100%	90	100%
	Mean ± SD	37.30 ± 7.08		36.73 ± 6.22		37.06 ± 6.50	
Gender	Male	13	43.3%	22	36.7%	35	38.9%
	Female	17	56.7%	38	63.3%	55	61.1%
	Total	30	100%	60	100%	90	100%

**Table 2:** Intragroup comparison of mean ± Std. Deviation of clinical parameters before and after 1 month of therapy in groups A and B using paired *t*-test.

Group	Index	Time	Mean	*N*	Std. deviation	t-test	df.	*P* value
Group A	PI	Before	2.127	30	0.177	36.546	29	*P* ≤ 0.001 (HS)
		After	0.533	30	0.184			
	GI	Before	2.103	30	0.133	59.024	29	*P* ≤ 0.001 (HS)
		After	0.618	30	0.081			
	BOP	Before	70.869	30	4.950	65.398	29	*P* ≤ 0.001 (HS)
		After	20.847	30	3.038			
	PPD	Before	4.650	30	0.544	18.259	29	*P* ≤ 0.001 (HS)
		After	3.067	30	0.173			
	CAL	Before	5.250	30	0.469	21.627	29	*P* ≤ 0.001 (HS)
		After	3.583	30	0.265			

Group B	PI	Before	2.017	30	0.102	40.835	29	*P* ≤ 0.001 (HS)
		After	0.693	30	0.141			
	GI	Before	2.047	30	0.183	35.066	29	*P* ≤ 0.001 (HS)
		After	0.925	30	0.104			
	BOP	Before	68.161	30	6.316	34.272	29	*P* ≤ 0.001 (HS)
		After	31.547	30	4.075			
	PPD	Before	4.617	30	0.409	22.321	29	*P* ≤ 0.001 (HS)
		After	3.233	30	0.254			
	CAL	Before	5.100	30	0.635	16.37	29	*P* ≤ 0.001 (HS)
		After	4.067	30	0.254			

*P* ≤ 0.05 was considered as significant.

**Table 3:** Intragroup comparison of mean ± Std. Deviation of serum Zn, Cu, and Mg levels at baseline before therapy and 1 month after therapy in both A and B groups using paired *t*-test.

Group	Index	Time	Mean	*N*	Std. deviation	*t*-test	df	*P* value
Group A	Mg	Before	1.540	30	0.081	-13.131	29	*P* ≤ 0.001 (HS)
		After	1.827	30	0.087			
	Zn	Before	130.532	30	33.892	-4.296	29	*P* ≤ 0.001 (HS)
		After	171.273	30	61.448			
	Cu	Before	199.364	30	32.004	7.109	29	*P* ≤ 0.001 (HS)
		After	178.747	30	31.504			

Group B	Mg	Before	1.540	30	0.067	-5.135	29	*P* ≤ 0.001 (HS)
		After	1.607	30	0.058			
	Zn	Before	97.667	30	6.031	-0.107	29	0.915 (NS)
		After	97.897	30	14.952			
	Cu	Before	186.577	30	34.583	3.775	29	0.001 (HS)
		After	159.444	30	12.792			

*P* ≤ 0.05 was considered as significant.

**Table 4:** Intragroup comparison of mean ± Std. Deviation of serum proinflammatory cytokine IL-1*β* and TNF-*α* levels at baseline before therapy and 1 month after therapy in both A and B groups using paired *t*-test.

Group	Index (pg/ml)	Time	Mean	*N*	Std. deviation	*t*-test	df	*P* value
Group A	IL-1*β*	Before	5.036	30	0.609	36.49	29	*P* ≤ 0.001 (HS)
		After	3.450	30	0.486		
	TNF-*α*	Before	10.561	30	0.713	25.234	29	*P* ≤ 0.001 (HS)
		After	7.374	30	0.652		

Group B	IL-1*β*	Before	10.561	30	0.606	7.114	29	*P* ≤ 0.001 (HS)
		After	4.534	30	0.529		
	TNF-*α*	Before	10.519	30	0.717	22.213	29	*P* ≤ 0.001 (HS)
		After	8.483	30	0.453		

*P* ≤ 0.05 was considered as significant.

**Table 5:** Intergroup comparison of curcumin (group A) and SRP groups (group B) after 1 month of therapy using unpaired *t*-test.

Parameters	Time	Group	*N*	Mean	Std. deviation	*t*-test	df	*P* value
PI	After therapy	Group A	30	0.533	0.184	-3.772	58	*P* ≤ 0.001
		Group B	30	0.693	0.141			(HS)
GI	After therapy	Group A	30	0.618	0.081	-12.746	58	*P* ≤ 0.001
		Group B	30	0.925	0.104			(HS)
BOP	After therapy	Group A	30	20.847	3.038	-11.53	58	*P* ≤ 0.001
		Group B	30	31.547	4.075			(HS)
PPD	After therapy	Group A	30	3.067	0.173	-2.973	58	0.004
		Group B	30	3.233	0.254			(HS)
CAL	After therapy	Group A	30	3.583	0.265	-4.316	58	*P* ≤ 0.001
		Group B	30	4.067	0.553			(HS)
Mg	After therapy	Group A	30	1.827	0.087	11.519	58	*P* ≤ 0.001
		Group B	30	1.607	0.058			(HS)
Zn	After therapy	Group A	30	171.273	61.448	6.355	58	*P* ≤ 0.001
		Group B	30	97.897	14.952			(HS)
Cu	After therapy	Group A	30	178.747	31.504	3.109	58	0.003
		Group B	30	159.444	12.792			(HS)
IL-1*β*	After therapy	Group A	30	3.450	0.486	-8.261	58	*P* ≤ 0.001
		Group B	30	4.534	0.529			(HS)
TNF-*α*	After therapy	Group A	30	7.374	0.652	-7.653	58	*P* ≤ 0.001
		Group B	30	8.483	0.453			(HS)

*P* ≤ 0.05 was considered as significant.

**Table 6:** Intergroup comparison of healthy control with chronic periodontitis A and B groups at baseline before therapy using unpaired *t*-test.

	Mean ± SD		*t*-test	df	*P* value	Sig.
Parameters	Control	Group A				
		Group B				
PI	0.100 ± 0.079	2.127 ± 0.178	57.161	58	*P* ≤ 0.001	(HS)
		2.017 ± 0.102	81.458	58	*P* ≤ 0.001	(HS)
GI	0.070 ± 0.092	2.103 ± 0.133	69.13	58	*P* ≤ 0.001	(HS)
		2.047 ± 0.183	52.945	58	*P* ≤ 0.001	(HS)
BOP	0.000 ± 0.000	70.869 ± 4.95	78.425	58	*P* ≤ 0.001	(HS)
		68.161 ± 6.316	59.108	58	*P* ≤ 0.001	(HS)
PPD	1.583 ± 0.708	4.650 ± 0.544	18.813	58	*P* ≤ 0.001	(HS)
		4.617 ± 0.409	20.322	58	*P* ≤ 0.001	(HS)
CAL	0.000 ± 0.000	5.250 ± 0.469	61.331	58	*P* ≤ 0.001	(HS)
		5.100 ± 0.635	43.978	58	*P* ≤ 0.001	(HS)
Zn	92.260 ± 13.510	130.532 ± 33.892	5.745	58	0.050	(S)
		97.667 ± 6.031	2.002	58	*P* ≤ 0.001	(HS)
Mg	1.880 ± 0.092	1.540 ± 0.081	-15.119	58	*P* ≤ 0.001	(HS)
		1.540 ± 0.067	-16.268	58	*P* ≤ 0.001	(HS)
Cu	52.847 ± 8.873	199.364 ± 32.004	24.164	58	*P* ≤ 0.001	(HS)
		186.577 ± 34.583	20.516	58	*P* ≤ 0.001	(HS)
IL-1*β*	3.120 ± 0.352	5.036 ± 0.609	14.909	58	*P* ≤ 0.001	(HS)
		4.951 ± 0.606	14.304	58	*P* ≤ 0.001	(HS)
TNF-*α*	7.188 ± 0.803	10.561 ± 0.713	17.205	58	*P* ≤ 0.001	(HS)
		10.519 ± 0.717	16.944	58	*P* ≤ 0.001	(HS)

*P* ≤ 0.05 was considered as significant.

**Table 7:** Intergroup comparison of healthy control with curcumin (A) and SRP groups (B) after 1 month of therapy using unpaired *t*-test.

	Mean ± SD		*t*-test	df	*P* value	Sig.
Parameters	Control	Group A				
		Group B				
PI	0.100 ± 0.079	0.533 ± 0.184	11.833	58	*P* ≤ 0.001	(HS)
		0.693 ± 0.141	20.092	58	*P* ≤ 0.001	(HS)
GI	0.070 ± 0.092	0.618 ± 0.081	24.573	58	*P* ≤ 0.001	(HS)
		0.925 ± 0.104	33.779	58	*P* ≤ 0.001	(HS)
BOP	0.000 ± 0.000	20.847 ± 3.038	37.585	58	*P* ≤ 0.001	(HS)
		31.547 ± 4.075	42.4	58	*P* ≤ 0.001	(HS)
PPD	1.583 ± 0.708	3.067 ± 0.173	11.146	58	*P* ≤ 0.001	(HS)
		3.233 ± 0.254	12.015	58	*P* ≤ 0.001	(HS)
CAL	0.000 ± 0.000	3.583 ± 0.265	73.97	58	*P* ≤ 0.001	(HS)
		4.067 ± 0.553	40.283	58	*P* ≤ 0.001	(HS)
Mg	1.880 ± 0.092	1.827 ± 0.087	-2.303	58	*P* ≤ 0.001	(S)
		1.607 ± 0.058	-13.693	58	*P* ≤ 0.001	(HS)
Zn	92.260 ± 13.510	171.273 ± 61.448	6.879	58	*P* ≤ 0.001	(HS)
		97.897 ± 14.952	1.532	58	0.131	(NS)
Cu	52.847 ± 8.873	178.747 ± 31.504	21.069	58	*P* ≤ 0.001	(HS)
	3.120 ± 0.352	159.444 ± 12.792	37.503	58	*P* ≤ 0.001	(HS)
IL-1*β*		3.45 ± 0.486	3.009	58	0.004	(HS)
	7.188 ± 0.803	4.534 ± 0.529	12.184	58	*P* ≤ 0.001	(HS)
TNF-*α*		7.374 ± 0.652	0.989	58	0.327	(NS)
		8.483 ± 0.453	7.695	58	*P* ≤ 0.001	(HS)

*P* ≤ 0.05 was accepted as statistically significant.

## Data Availability

The data used to support the findings of this study are available from the corresponding author upon request.
